# Eco-epidemiology of rodent-associated trombiculid mites and infection with *Orientia* spp. in Southern Chile

**DOI:** 10.1371/journal.pntd.0011051

**Published:** 2023-01-12

**Authors:** María Carolina Silva de la Fuente, Caricia Pérez, Constanza Martínez-Valdebenito, Ruth Pérez, Cecilia Vial, Alexandr Stekolnikov, Katia Abarca, Thomas Weitzel, Gerardo Acosta-Jamett

**Affiliations:** 1 Instituto de Medicina Preventiva Veterinaria and Center for Disease Surveillance and Evolution of Infectious Diseases, Facultad de Ciencias Veterinarias, Universidad Austral de Chile, Valdivia, Chile; 2 Facultad de Ciencias Agrarias y Forestales, Departamento de Ciencias Agrarias, Universidad Católica del Maule, Talca, Chile; 3 Instituto de Ciencias e Innovación en Medicina (ICIM), Facultad de Medicina Clínica Alemana, Universidad del Desarrollo, Santiago, Chile; 4 Departamento de Enfermedades Infecciosas e Inmunología Pediátricas, Escuela de Medicina, Pontificia Universidad Católica de Chile, Santiago, Chile; 5 Laboratorio de Infectología y Virología Molecular, Red Salud UC–Christus, Santiago, Chile; 6 Zoological Institute of the Russian Academy of Sciences, Saint Petersburg, Russia; 7 Laboratorio Clínico, Clínica Alemana de Santiago, Facultad de Medicina Clínica Alemana, Universidad del Desarrollo, Santiago, Chile; Centers for Disease Control and Prevention, UNITED STATES

## Abstract

**Background:**

Scrub typhus is a potentially severe infection caused by bacteria of the genus *Orientia*, endemic in Asia-Pacific and recently discovered in southern Chile. The presented study aimed to determine the prevalence and species richness of rodent-associated trombiculid mites and their infection with *Orientia* spp. in different areas of two regions in southern Chile.

**Methodology/Principal findings:**

During summer 2020, trombiculid mites were collected from rodents captured in three areas in southern Chile known to be endemic for scrub typhus (Cochamó and Chiloé Island in the Los Lagos Region and Tortel in the Aysén Region). A total of 132 rodents belonging to five species were captured using Sherman-like traps; 89.4% were infested with trombiculids. Mite specimens were morphologically identified and subsequently tested by *Orientia*-specific qPCR. Six mite species were identified. Among chigger-infested rodents, 33.9% carried *Orientia*-positive mites; this rate was higher in Tortel (63.8%) than in Cochamó (45.0%) and Chiloé Island (2.0%). The analysis of individual mites (n = 901) revealed that 31.2% of *Herpetacarus antarctica* samples (n = 202) were positive for *Orientia* DNA; the prevalence was 7.0% in *Paratrombicula neuquenensis* (n = 213), 6.9% in *Herpetacarus eloisae* (n = 144), 3.6% in *Argentinacarus expansus* (n = 55), and 0% in *Paratrombicula goffi* (n = 110) and *Quadraseta chiloensis* (n = 177). The southernmost site (Tortel) showed the highest rates of trombiculid infestation, trombiculid load, and *Orientia* infection in the captured rodents.

**Conclusions/Significance:**

Our study provides new insights into the trombiculid fauna and prevalence of *Orientia* in mites collected from wild rodents in southern Chile. *Orientia* DNA was detected in four of the six mite species. Rates of infestation, mite loads, and *Orientia* prevalences differed geographically and were highest in the Aysén Region. Our data improve our knowledge on possible vectors of scrub typhus and their distribution in Chile.

## Author summary

Scrub typhus is an emerging zoonotic infection caused by *Orientia* species in Asia-Pacific and recently discovered in Chile. The disease is transmitted by chigger mites; however, the knowledge on the vectors in Chile is scarce. We undertook a field study to capture wild rodents in three areas in southern Chile and to determine their infestation with chiggers infected with the bacteria. The study revealed that 89.4% of rodents carried trombiculid mites belonging to six species. The mite fauna and prevalence of *Orientia* showed geographical variations. *Orientia* DNA was found in four chigger species, mainly in two species of the genus *Herpetacarus*. The southernmost study area showed the highest prevalence of trombiculid infestation and infection with *Orientia* and must be considered a hot spot for scrub typhus in Chile. This study provides important new knowledge on the eco-epidemiology of scrub typhus in Chile.

## Introduction

Scrub typhus threatens over one billion people in the Asia-Pacific region, where, despite its high morbidity and mortality, it is considered a neglected disease [1]. Initially, scrub typhus was thought to be restricted to an area known as “tsutsugamushi triangle”; however, the clinical cases reported from South America and the Middle East in the last 15 years, together with serological evidence from Africa and Latin America, suggest a much wider distribution of the disease [2]. Recent data from Chile showed that endemic areas included various geographical and climatic regions over a distance of almost 2,000 km [3–5].

Scrub typhus is caused by obligate intracellular bacteria of the genus *Orientia*, belonging to the Rickettsiacea family [6], with three species described so far, *Orientia tsutsugamushi* occurring in Asia-Pacific, *Candidatus* Orientia chuto (from United Arab Emirates), and *Candidatus* Orientia chiloensis (from Chile) [7,8]. *O*. *tsutsugamushi* is transmitted by mites of the family Trombiculidae (Acari: Trombidiformes), mostly belonging to the genus *Leptotrombidium*, which, through transovarial and transstadial transmission, also serve as the pathogen’s reservoir [9]. The mites’ ectoparasitic larvae, called chiggers, have low host specificity [10], but rodents seem to be the main determinants for the maintenance of stable mite populations in Asia-Pacific [11,12]. In Chile, the knowledge of the eco-epidemiological aspects of scrub typhus and its vectors is incomplete. Up to now, 25 trombiculid mite species of 12 genera have been recorded in Chile, mainly in relation to reptiles [13–16]. A first investigation of rodent-associated mites from scrub typhus endemic localities on Chiloé Island reported three chigger species, of which two were new to science; one novel species, *Herpetacarus eloisae*, was found positive for *Orientia* DNA, suggesting its capacity as vector and reservoir of scrub typhus [16,17]. In addition, a recent report proved *Herpetacarus antarctica* as vector of scrub typhus in the Aysén Region in southern Chile [18].

The here presented study analyzed the rodent-associated trombiculid fauna and the possible role of trombiculids as vectors and reservoirs of *Orientia* spp. in three areas with endemic scrub typhus in southern Chile.

## Methods

### Ethics statement

The study was approved by the Scientific Ethics Committee for the Care of Animals and the Environment of Pontificia Universidad Católica de Chile (N° 160816007, January 28, 2020) and by the Agricultural and Livestock Service (SAG) (Exempt Resolution No.: 858/2020, February 3, 2020).

### Study sites

**Fig 1:**
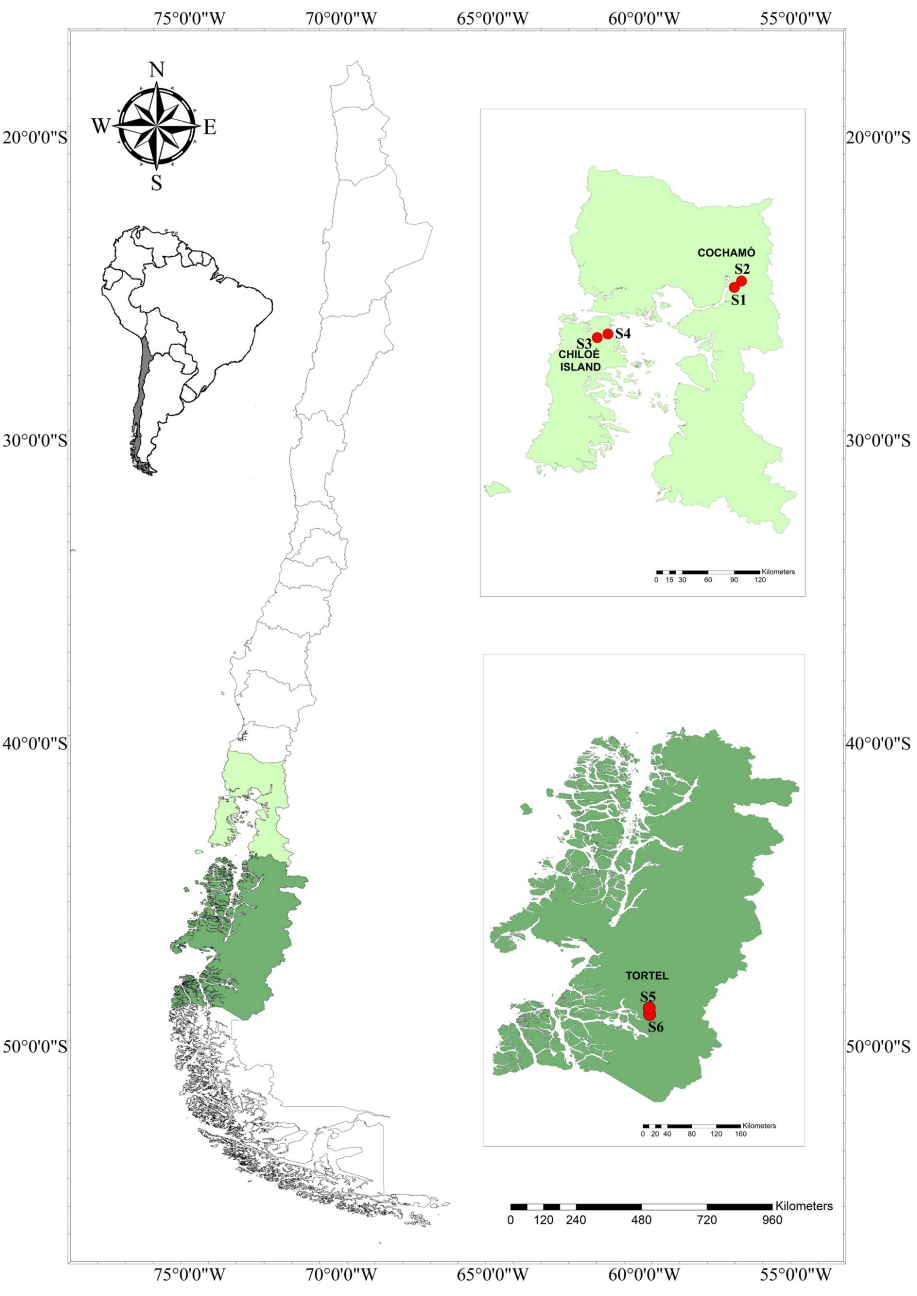
Map of Chile indicating the study areas. Sites S1 and S2 in Cochamó, S3 and S4 on Chiloé Island in Los Lagos Region (light green) and S5 and S6 in Tortel in Aysén Region (dark green) are highlighted. (Map made in QGIS Geographic Information System. Open Source Geospatial Foundation Project. http://qgis.osgeo.org. Shapes downloaded from an open source from the Biblioteca del Congreso Nacional, Available at https://www.bcn.cl/siit/mapas_vectoriales/index_html).

The study was carried out in three areas in southern Chile, Cochamó (41°45’40”S, 72°5’35”W), Chiloé Island (41°52’15”S, 73°48’58”W), both in the Los Lagos Region, and Tortel (47°47’54”S, 73°32’24”W) located in the Aysén Region (Fig 1). In all three study areas cases of scrub typhus have been detected by our research group [4,19]. Cochamó has a temperate oceanic climate, with an annual rainfall of 3,407mm and an average annual temperature in the Andean valleys of 6.6°C; however, in summer, average temperature raises up to 13.2°C and rainfall decreases [20]. In Cochamó the two capture sites (S1 and S2) are covered by Valdivian temperate rainforest with a certain degree of human intervention such as thinning and construction sites [20]. Chiloé Island also has temperate oceanic climate with an annual rainfall of 2,090 mm and average annual temperature of 12°C [21]. The landscape consists of fragments of Valdivian temperate rainforest of different sizes, shapes, degrees of isolation and degradation. The selected capture sites (S3 and S4) correspond to localities N°3 and N°4, respectively, of our previous study [17]. Tortel has a subpolar oceanic climate (cold temperate, rainy, coastal), annual rainfall of 3,500–4,000 mm, and average temperature of 6°C–8°C. In this area, sampling was carried out at two sites, Aumén (S5) and Laguna Caiquenes (S6), about 18km southeast of the village Caleta Tortel. The vegetation consists of Valdivian temperate rainforest with bushy thicket with a lower floor of hardy perennial grassland [22].

### Rodent trapping

**Fig 2:**
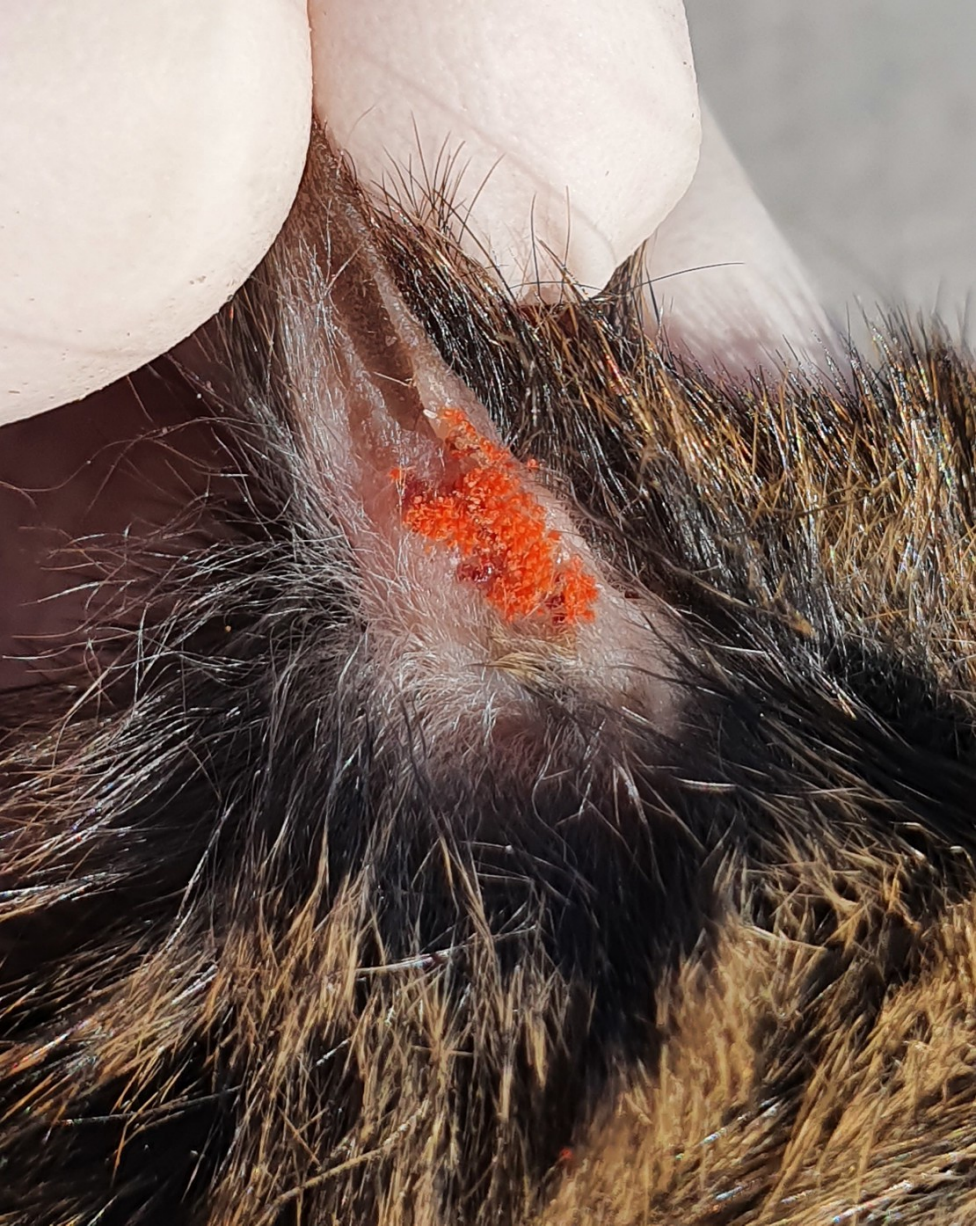
Trombiculid mites (*Herpetacarus antarctica*) at the base of the ear of *Loxodontomys micropus* from Tortel area.

Rodent sampling was carried out during end of February and beginning of March 2020, within the austral summer period. In each study area, two sampling sites were surveyed. At each site, between 75–80 Sherman-type traps (300 × 100 × 110 mm) were activated at 5 meters distance, placed under bushes or fallen logs or in burrows. Traps were placed simultaneously in each site during 5 consecutive nights with an effort of 375–400 trap-nights per site, 750–800 trap-nights per area, and total of 2,350 trap-nights. Captured rodents were transferred to a central processing tent, where they were chemically immobilized in an induction chamber containing cotton soaked with isoflurane (USP, Baxter; 1 mL of isoflurane per 500 mL of chamber volume) [17]. After sedation, male and juvenile rodents (both sexes) were euthanized by cervical dislocation [23], while adult females were released at the capture site after examination. Each rodent was measured with a caliper (Serie 500, Mitutoyo, Japan), weighed (Spring Scale 10100, Pesola, Switzerland), and morphologically identified, as previously described [24]. Subsequently, the fur of each rodent was inspected using fine-tipped tweezers to collect trombiculid mites, which were mostly located in the ears (Fig 2). Trombiculids from individual rodents were stored in vials with 96% ethanol for subsequent taxonomic and molecular analyses. Rodent’s tissues (i.e. liver, spleen and lungs) were also obtained and deposited in 96% ethanol for further molecular studies.

Due to the study team’s exposure risk to Andes virus, causing hantavirus cardiopulmonary syndrome [25], rodents were handled following biosafety recommendations of the CDC and the American Society of Mammalogists [26]. Thus, researchers were equipped with a full-face respirators (6000 Series, 3M, Minnesota, USA) equipped with P100 ultrafine particle filters (2000 Series, 3M), disposable coveralls, shoe covers, and latex gloves. Traps were disinfected daily with hypochlorite solution. In addition, members of the fieldwork team were advised and followed-up for 5 weeks after the field period to recognize possible signs and symptoms of scrub typhus [27].

### Mite identification

**Fig 3:**
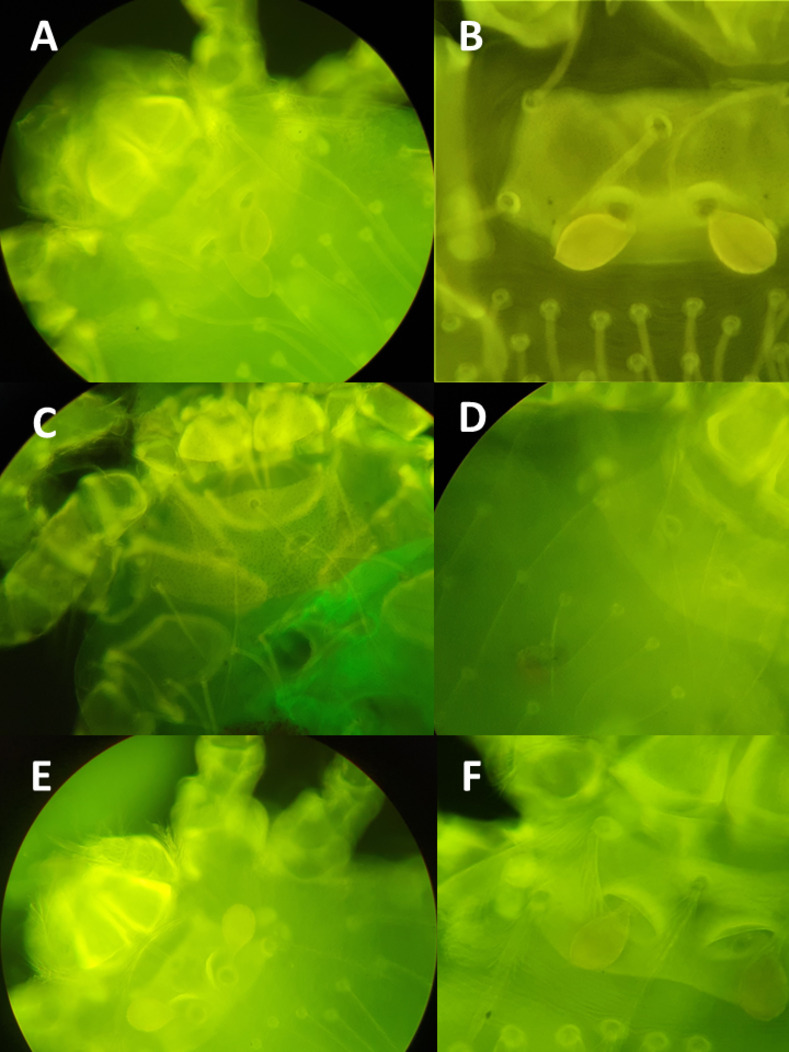
Trombiculids observed with an epifluorescence microscope prior to molecular analysis. A. *Herpetacarus eloisae*. B. *Herpetacarus antarctica*. C. *Paratrombicula goffi*. D. *Paratrombicula neuquenensis*. E. *Quadraseta chiloensis*. F. *Argentinacarus expansus*.

The taxonomic analyses of mite specimens were carried out at the Instituto de Medicina Preventiva Veterinaria, Universidad Austral de Chile in Valdivia, Chile. Initially, mites were observed under a stereo microscope (SZ61 Olympus, Tokyo, Japan), which permitted to separate different morphotypes. Approximately 20 samples of each morphotype were rinsed with Nesbitt solution and individually mounted with Berlese medium between a microscope slide and coverslip [28]. Mounted specimens were further analyzed under an optical microscope (BS-2030T, BestScope, Beijing, China) with a magnification of 640×, following keys proposed by Brennan and Goff [29] as well as Stekolnikov and González-Acuña [13]. The remaining samples were examined under a fluorescence optical microscope (BS-2030FT, Bestscope), which allowed to observe species-specific morphological criteria, e.g. shape of the scutum and its setae (Fig 3), as previously described [30]. This permitted to separate and store individual and pooled mites of known species for further molecular analyses. A subset of mounted samples of each mite species was sent to the Zoological Institute of the Russian Academy of Sciences in Saint Petersburg, Russia, to confirm the morphological identification.

### Molecular detection of *Orientia* DNA in trombiculid mites

With the exception of mounted specimens, a subset of mites was tested for the presence of *Orientia* DNA as individuals or as pools. Of each chigger species (collected from an individual rodent) approximately 10 samples were tested as individual mites, while the remaining specimens were analyzed as pools of 3–60 mites in Cochamó and Chiloé Island and of 10–100 mites in Tortel. Individual and pooled samples were dried at 72°C for 3 hours in an oven to eliminate excess ethanol and stored at -80°C in a mixture of 180 μL of lysis buffer and 20 μL of proteinase K (Qiagen, Hilden, Germany). To mechanically disrupt the mites’ exoskeletons, we used a thermal method, based on previously described protocols [31,32]. Samples were thawed at room temperature and then subjected to three freeze-thaw cycles (2 minutes in liquid nitrogen and 2 minutes at 70°C), followed by incubation at 56°C for 3 hours in a thermal bath. After this procedure, DNA was extracted using the manufacturer’s instructions for the QIAamp DNA Mini Kit (Qiagen). Finally, samples were eluted in 50 μL of buffer AE (Qiagen) and stored at 4°C, if molecular testing was carried out within 24 hours, or at -80°C, if this was done later. For the molecular detection of *Orientia* DNA, real-time quantitative polymerase chain reaction (qPCR) targeting the *rrs* gene (16S RNA) was used, which has recently been developed to detect *Orientia* species including strains from Chile [33].

### Data analysis

Firstly, we analyzed the pattern of trombiculid infestation on the rodent species captured in different sites, which included infestation rates, mean abundance per host (i.e. chiggers index), and mean species richness per host. Secondly, we assessed whether the number of chigger species and the total load of chiggers parasitizing each host differed between areas using Kruskal Wallis test and Dunn’s Test for post-hoc comparisons. Finally, we performed chi-square test and Bonferroni adjustment for post-hoc analyses to compare *Orientia*-positive mite rates in wild rodents between host species and sampling areas. All statistical analyses were performed using R software [34].

## Results

A total of 132 rodents were captured, 28 (21.2%) in Cochamó, 57 (43.2%) on Chiloé Island, and 47 (35.6%) in Tortel (Table 1). Rodents belonged to five species, *Abrothrix olivacea* (50, 37.9%), *Oligoryzomys longicaudatus* (43, 32.6%), *Loxodontomys micropus* (25, 18.9%), *Geoxus valdivianus* (11, 8.3%), and *Abrothrix manni* (3, 2.3%). As shown in Table 1, the species composition and abundance varied between the study areas and sites, with *O*. *longicaudatus* being the only species occurring in all three study areas.

**Table 1:** Summary of wild rodent species and prevalence of chigger mites in each sampling area.

Sites and host species	N° of rodents collected (% of total)	N° of rodents infested with mites (prevalence %)	N° of mites collected (% from total mites per area)	Chigger index (no. mites/no. rodents)
**Cochamó**	**28 (21.2)**	**20 (71.4)**	**404 (4.2)**	**14.4**
**Site 1**	**9 (32.1)**	**6 (66.7)**	**42 (10.4)**	**4.7**
*Abrothrix olivacea*	9 (100)	6 (66.7)	42 (100)	4.7
**Site 2**	**19 (67.9)**	**14 (73.7)**	**362 (89.6)**	**19.1**
*Abrothrix olivacea*	9 (47.4)	7 (77.7)	241 (66.6)	26.6
*Abrothrix manni*	3 (15.8)	3 (100)	42 (11.6)	14.0
*Oligoryzomys longicaudatus*	7 (36.8)	4 (57.1)	79 (21.8)	11.3
**Chiloé Island**	**57 (43.2)**	**51 (89.5)**	**1,100 (11.4)**	**19.3**
**Site 3**	**46 (80.7)**	**41 (89.1)**	**910 (82.7)**	**19.7**
*Abrothrix olivacea*	24 (52.2)	20 (83.3)	413 (45.4)	17.2
*Geoxus valdivianus*	10 (21.7)	10 (100)	222 (24.4)	22.2
*Oligoryzomys longicaudatus*	12 (26.1)	11 (91.7)	275 (30.2)	22.9
**Site 4**	**11 (19.3)**	**10 (90.9)**	**190 (17.3)**	**17.3**
*Abrothrix olivacea*	8 (72.7)	7 (87.5)	149 (78.4)	18.6
*Geoxus valdivianus*	1 (9.1)	1 (100)	25 (13.2)	25.0
*Oligoryzomys longicaudatus*	2 (18.2)	2 (100)	16 (8.4)	8.0
**Tortel**	**47 (35.6)**	**47 (100)**	**8,186 (84.1)**	**174.2**
**Site 5**	**11 (23.4)**	**11 (100)**	**2,350 (28.7)**	**213.6**
*Loxodontomys micropus*	8 (53.2)	8 (100)	2,009 (85.5)	251.1
*Oligoryzomys longicaudatus*	3 (46.8)	3 (100)	341 (14.5)	113.7
**Site 6**	**36 (76.6)**	**36 (100)**	**5,836 (71.3)**	**162.1**
*Loxodontomys micropus*	17 (47.2)	17 (100)	2,985 (51.1)	175.6
*Oligoryzomys longicaudatus*	19 (52.8)	19 (100)	2,851 (48.9)	150.1
**Total**	**132**	**118 (89.4)**	**9,690**	**73.4**

### Chigger infestation

Overall, 118 (89.4%) of the 132 captured rodents were infested with chigger mites (Table 1). Trombiculid mites affected all rodent species; although infestation rates differed and were highest on *A*. *manni* (3/3, 100%), *G*. *valdivianus* (11/11, 100%), and *L*. *micropus* (25/25, 100%), followed by *O*. *longicaudatus* (39/43, 90.1%) and *A*. *olivacea* (40/50, 80.0%); these differences were not statistically significant (p>0.05). The total ectoparasitic load was 9,690 mites, with an overall mean chigger index of mites per rodent of 73.4 (range 4.7–251.1 per rodent species per site). Chigger infestation rates on the captured rodents varied significantly by study area. Cochamó had the lowest prevalence of trombiculids (404 samples; chigger index 14.4); all rodent species were infested with chigger index rates of 4.7 to 26.6 (Table 1). Chiloé Island had a slightly higher prevalence of trombiculid mites (1,100 specimens, chigger index 19.3). The chigger prevalence was highest in Tortel, where a total of 8,186 mites were collected and the chigger index was 174.2. In this area, the infestation rate was 100% in both sites; the highest chigger index was recorded on *L*. *micropus* with 251.1 and 175.6 in sites 5 and 6, respectively (Table 1).

Among the 9,690 collected chigger specimens we identified six species. Four of them (*Herpetacarus antarctica*, *Herpetacarus eloisae*, *Paratrombicula goffi*, and *Quadraseta chiloensis*) were previously known from Chile and two (*Paratrombicula neuquenensis* and *Argentinacarus expansus*) were previously known only from their type series collected from rodents in Argentina [35]. Overall, rodents were most frequently infested by *H*. *antarctica* (39.8%), followed by *Q*. *chiloensis* (35.6%), *P*. *goffi* (28.0%), *H*. *eloisae* (24.6%), *P*. *neuquenensis* (16.1%), and *A*. *expansus* (7.6%) (Table 2). With 8,426 (87.0%) specimens, *Herpetacarus* represented the predominant genus, followed by *Quadraseta* with 721 specimens (7.4%), and *Paratrombicula* (488 specimens; 5.0%), while 55 samples (0.6%) belonged to *Argentinacarus*. The chigger fauna displayed geographical variations, with four species in Cochamó and Chiloé Island, and a single species, *H*. *antarctica*, in Tortel (Table 2). The mite load per rodent varied from 0 to 534 chiggers; *L*. *micropus* captured in Tortel and infested with *H*. *antarctica* presented the highest median abundance (282.5 and 155 in sites 5 and 6, respectively). On the contrary, rodents on Chiloé Island infested with *A*. *expansus* had the lowest median abundance (Table 2).

**Table 2:** Median abundance of chiggers of different species from wild rodents. In parenthesis is the percentage of rodents parasitized by the respective mite species.

Sites and host species	n	*Herpetacarus antarctica*	*Herpetacarus eloisae*	*Paratrombicula goffi*	*Paratrombicula neuquenensis*	*Quadraseta chiloensis*	*Argentinacarus expansus*
**Cochamó**	**20**	**0**	**5.0 (60.0)**	**0**	**6.0 (95.0)**	**8.5 (10.0)**	**7.0 (25.0)**
**Site 1**	**6**	**0**	**4.0 (16.7)**	**0**	**3.0 (83.3)**	**0**	**7.0 (16.7)**
*Abrothrix olivacea*	6	0	4.0 (16.7)	0	3.0 (83.3)	0	7.0 (16.7)
**Site 2**	**14**	**0**	**6.0 (78.6)**	**0**	**6.5 (100)**	**8.5 (14.3)**	**9.0 (28.6)**
*Abrothrix olivacea*	7	0	7.0 (57.1)	0	10.0 (100)	8.5 (28.6)	6.0 (42.9)
*Abrothrix manni*	3	0	6.0 (100)	0	3.0 (100)	0	12.0 (33.3)
*Oligoryzomys longicaudatus*	4	0	6.5 (100)	0	4.5 (100)	0	0
**Chiloé Island**	**51**	**0**	**3.0 (33.3)**	**3.0 (64.7)**	**0**	**14.0 (78.4)**	**2.5 (7.8)**
**Site 3**	**41**	**0**	**2.5 (29.6)**	**3.0 (76.6)**	**0**	**15.0 (74.1)**	**2.0 (4.9)**
*Abrothrix olivacea*	20	0	2.0 (10.0)	3.0 (75.0)	0	17.0 (75.0)	2.0 (5.0)
*Geoxus valdivianus*	10	0	10.0 (50.0)	5.0 (90.0)	0	3.5 (60.0)	2.0 (10.0)
*Oligoryzomys longicaudatus*	11	0	2.5 (18.2)	2.0 (63.6)	0	16.0 (100)	0
**Site 4**	**10**	**0**	**5.0 (70.0)**	**3.5 (20.0)**	**0**	**7.0 (80.0)**	**3.0 (20.0)**
*Abrothrix olivacea*	7	0	5.0 (71.4)	3.5 (28.9)	0	14.0 (71.4)	3.0 (28.6)
*Geoxus valdivianus*	1	0	22.0 (100)	0	0	3.0 (100)	0
*Oligoryzomys longicaudatus*	2	0	2.0 (50.0)	0	0	7.0 (100)	0
***Tortel***	**47**	**140.0 (100)**	**0**	**0**	**0**	**0**	**0**
**Site 5**	**11**	**247.0 (100)**	**0**	**0**	**0**	**0**	**0**
*Loxodontomys micropus*	8	282.5 (100)	0	0	0	0	0
*Oligoryzomys longicaudatus*	3	54.0 (100)	0	0	0	0	0
**Site 6**	**36**	**128.5 (100)**	**0**	**0**	**0**	**0**	**0**
*Loxodontomys micropus*	17	155.0 (100)	0	0	0	0	0
*Oligoryzomys longicaudatus*	19	95.0 (100)	0	0	0	0	0
**Total**	**118**	**140.0 (39.8)**	**4.0 (24.6)**	**3.0 (28.0)**	**6.0 (16.1)**	**13.0 (35.6)**	**5.0 (7.6)**

Most of the infested rodents (60%) were parasitized my more than one chigger species. The number of trombiculid species per rodent (species richness) varied significantly among the areas (Kruskal-Wallis chi-squared = 17.5, p<0.001), with a higher median richness in animals from Cochamó and Chiloé Island (2 chigger species/rodent) than in those from Tortel (1 chigger species/rodent, Dunn’s Test, p<0.05). The chigger loads per rodent also displayed significant geographical variations (Kruskal-Wallis chi-squared = 68.0, p<0.001), with animals from Tortel presenting higher median load (140 chiggers/rodent) than those from Cochamó and Chiloé Island (7 and 11 chiggers/rodent, respectively, Dunn’s Test, p<0.05).

### Testing of mites for *Orientia* spp. DNA

According to the analysis of both individual and pooled mites, 33.9% (40/118) of mite-infested rodents carried *Orientia*-positive chiggers with statistically significant differences between areas (χ^2^ = 43.1, d.f. = 2, p<0.001). With 2.0% (1/51), rodents captured on Chiloé Island showed a significantly lower rate (p<0.05) than those from Tortel (63.8%, 30/47) and from Cochamó (45.0%, 9/20); the difference between the two latter areas was not significant (p>0.05) (Table 3).

**Table 3:** Prevalence of infection with *Orientia* spp. in trombiculid specimens of different species, collected from wild rodents.

Sites and host species	Prevalence of rodents with *Orientia-*positive mites (infected/total)*	Prevalence of *Orientia*-positive mites (infected/total)**						
		*Herpetacarus antarctica*	*Herpetacarus eloisae*	*Paratrombicula goffi*	*Paratrombicula neuquenensis*	*Quadraseta chiloensis*	*Argentinacarus expansus*	Total
**Cochamó**	**45.0 (9/20)**	–	**10.2 (9/88)**	–	**7.0 (15/213)**	**0 (0/9)**	**4.7 (2/43)**	**7.4 (26/353)**
**Site 1**	**33.3 (2/6)**	–	**0 (0/3)**	–	**24.1(7/29)**	**0 (0/4)**	–	**19.4 (7/36)**
*Abrothrix olivacea*	33.3 (2/6)	–	0 (0/3)	–	24.1 (7/29)	0 (0/4)	–	19.4 (7/36)
**Site 2**	**50.0 (7/14)**	–	**10.6 (9/85)**	–	**4.4 (8/184)**	0 (0/5)	**4.7 (2/43)**	**5.9 (19/321)**
*Abrothirx olivacea*	42.9 (3/7)	–	6.8 (3/44)	–	0.8 (1/132)	0 (0/5)	6.1 (2/33)	2.8 (6/218)
*Abrothrix manni*	33.3 (1/3)	–	14.3 (2/14)	–	7.7 (1/13)	–	0 (0/10)	8.1 (3/37)
*Oligoryzomys longicaudatus*	75.0 (3/4)	–	14.8 (4/27)	–	15.4 (6/39)	–	–	15.2 (10/66)
**Chiloé Island**	**2.0 (1/51)**	–	**1.8 (1/56)**	**0 (0/110)**	–	**0 (0/168)**	**0 (0/12)**	**0.3 (1/346)**
**Site 3**	**2.4 (1/41)**	–	**3.4 (1/29)**	**0 (0/103)**	–	**0 (0/136)**	**0 (0/6)**	**0.4 (1/274)**
*Abrothrix olivacea*	5.0 (1/20)	–	16.7 (1/6)	0 (0/50)	–	0 (0/71)	0 (0/4)	0.8 (1/131)
*Geoxus valdivianus*	0 (0/10)	–	0 (0/18)	0 (0/33)	–	0 (0/19)	0 (0/2)	0 (0/72)
*Oligoryzomys longicaudatus*	0 (0/11)	–	0 (0/5)	0 (0/20)	–	0 (0/46)	–	0 (0/71)
**Site 4**	**0 (0/10)**	–	**0 (0/27)**	**0 (0/7)**	–	**0 (0/32)**	**0 (0/6)**	**0 (0/72)**
*Abrothrix olivacea*	0 (0/7)	–	0 (0/20)	0 (0/7)	–	0 (0/20)	0 (0/6)	0 (0/53)
*Geoxus valdivianus*	0 (0/1)	–	0 (0/5)	–	–	0 (0/3)	–	0 (0/8)
*Oligoryzomys longicaudatus*	0 (0/2)	–	0 (0/2)	–	–	0 (0/9)	–	0 (0/11)
**Tortel**	**63.8 (30/47)**	**31.2 (63/202)**	–	–	–	–	–	**31.2 (63/202)**
**Site 5**	**81.8 (9/11)**	**47.5 (47/99)**	–	–	–	–	–	**47.5 (47/99)**
*Loxodontomys micropus*	75.0 (6/8)	47.6 (39/82)	–	–	–	–	–	47.6 (39/82)
*Oligoryzomys longicaudatus*	100 (3/3)	47.1 (8/17)	–	–	–	–	–	47.1 (8/17)
**Site 6**	**58.3 (21/36)**	**15.5 (16/103)**	–	–	–	–	–	**15.5 (16/103)**
*Loxodontomys micropus*	70.6 (12/17)	12.9 (8/62)	–	–	–	–	–	12.9 (8/62)
*Oligoryzomys longicaudatus*	47.4 (9/19)	19.5 (8/41)	–	–	–	–	–	19.5 (8/41)
**Total**	**33.9 (40/118)**	**31.2 (63/202)**	**6.9 (10/144)**	**0 (0/110)**	**7.0 (15/213)**	**0 (0/177)**	**3.6 (2/55)**	**10.0 (90/901)**

*Individual chigger specimens and chigger pools; **Individual chigger specimens

To determine the prevalence of infection with *Orientia* spp. within the collected mite population, 901 individual specimens were analyzed by *Orientia*-specific qPCR. Overall, *Orientia* was detected in 90 (10.0%) of samples, with prevalences of 31.2% in *H*. *antarctica*, 7.0% in *P*. *neuquenensis*, 6.9% in *H*. *eloisae*, and 3.6% in *A*. *expansus* (Table 3). The highest prevalence was detected in Tortel, where 31.2% of mites (all *H*. *antarctica*) were infected (Table 3). In Cochamó, 7.4% of mite samples were *Orientia*-positive, with species-specific rates of 10.2%, 7.0%, 4.7%, and 0% for *H*. *eloisae*, *P*. *neuquenensis*, *A*. *expansus*, and *Q*. *chiloensis*, respectively. On Chiloé Island, the overall rate was the lowest (0.3%) and *Orientia* DNA was only detected in *H*. *eloisae* (1.8%), but not in other mite species (*P*.*goffi*, *Q*. *chiloensis*, and *A*. *expansus*) (Table 3). In neither locality, positivity rates of individual mites were significantly affected by the rodent species, from which mites were collected (p>0.05). The geographical differences of *Orientia* prevalence in mites, however, were significant (χ^2^ = 139.9, d.f. = 2, p<0.001), with a highest rate in Tortel and lowest rate on Chiloé Island (p<0.05).

## Discussion

Trombiculid mites are important but neglected vectors of rickettsial infections and other pathogens [36]. In the Asia-Pacific region, *Leptotrombidium* species serve as vectors of *O*. *tsutsugamushi* [9]. First data from Sub-Saharan Africa demonstrated that rodent-associated chiggers in Kenya, belonging to the genera *Neotrombicula* and *Microtrombicula*, contained DNA sequences closely resembling those of *Ca*. O. chuto [37]; in this region, however, clinical cases of scrub typhus have yet not been reported. In Chile, the knowledge of the trombiculid mite fauna and its possible role in the transmission of the recently discovered third *Orientia* species (*Ca*. O. chiloensis) is scarce. A first field study on Chiloé Island in 2018 found one known and two new rodent-associated chigger species; one of the novel species (*H*. *eloisae*) was infected with *Orientia* [17]. Finally, a recent report described chiggers collected from humans and environment in Tortel, providing first evidence that *H*. *antarctica* serves as vector and reservoir of scrub typhus [18].

Most information on the ecology of the vectors of scrub typhus derives from Asia-Pacific [12]. In this region, the species richness of trombiculids is higher in areas of mid-latitudes and decreases in higher latitudes [10,38,39]. The present study suggests a similar gradient in Chile, as a total of five species of trombiculids were found in the Los Lagos Region, while only a single species was identified in the more southern Aysén Region. Chigger infestation rates of different wild rodent species detected in our study ranged from 57.1% to 100%, which is compatible to reports in Asia-Pacific [12]. As reported from other regions, we saw no host specificity, as larval mites feed opportunistically on a variety of hosts [40–42]. A recent systematic review of the ecology of scrub typhus by Elliot et al. [12], found that 16% of studies reported chigger index values of infested rodents. Our results from Cochamó and Chiloé Island are within the range of these studies; however, the values detected on *L*. *micropus* in the two sites of Tortel (i.e. 251.1 and 175.6 mites/rodent) are among the highest worldwide [12].

In Asia-Pacific the spatial distribution of chiggers is heterogeneous, with distinct areas of high mite density, so called “mite islands” [12,43]. According to Chakraborty and Sarma [44], various environments including sandy beaches, mountains, rainforests, riverbanks, and grass lands might provide optimal conditions for mite proliferation. The resulting higher prevalence and richness of chigger mites have been associated to seasonal scrub typhus outbreaks [45]. Such favorable conditions for chigger proliferation might also be present in our study areas, which all represented sites, where clinical scrub typhus cases had been recorded in previous years. The high infestation rates and chigger index values in some of the study sites support the concept of mite islands within the Chilean chigger ecosystems; however, future ecological studies are needed to assess whether a combination of factors such as extreme temperatures in summer months could be favoring the life cycle of chiggers and the bacteria over a wider spatial range around possible hot spots. Apart from the noted differences in mite fauna and density, the infection rates with *Orientia* varied significantly. The mite abundance and *Orientia* infection rate was especially striking in Tortel. As mentioned above, our field researchers recorded multiple mite bites and one suffered from scrub typhus; unengorged mites were also ubiquitous noted on lower vegetation [18]. In addition, the *Orientia* infection rate of individual mites in this area was 24-times and 100-times higher than in Cochamó and Chiloé Island (Table 3), respectively. Our findings in Chiloé Island might have been influenced by temporal aspects or sampling error, such as changes in temperature and humidity among years and reduced trapping effort, which might be responsible for the lower captured rodents and *Orientia* infection in mites between the here reported results and a previous field project in 2018 at similar localities [17]. Similar annual variations of mite species have been described in the Asia-Pacific [46–48]. Further studies are necessary to understand these fluctuations, which could also be caused by variations of the rodent population or anthropogenic changes in the habitat.

According to a review from 2019, about 50 of the >3000 known species of Trombiculidae worldwide are known to be positive to *Orientia* and; among those, approximately 15 species, all belonging to the genus *Leptotrombidium* are confirmed or possible vectors of scrub typhus [12]. The cited review does not include recent data from Chile, according to which *Orientia* DNA was found in a novel trombiculid species, *H*. *eloisae*, parasitizing rodents collected on Chiloé Island, while two other mite species, *P*. *goffi* and *Q*. *chiloensis*, tested negative [16,17]. The present study confirmed this; among the four species collected in this area, only *H*. *eloisae* carried *Orientia* DNA. The report from 2022 described *Orientia* DNA in *H*. *antarctica* samples, which were collected from a member of the research team in Tortel, who later developed scrub typhus, thus providing first direct evidence of the scrub typhus transmission by a trombiculid mite not belonging to the genus *Leptotrombidium* [18]. The here presented study detected the highest *Orientia* infection rate in this species. As *H*. *antarctica* has been described in the Magallanes Region in southern Chile [13], it is probably the vector in Tierra del Fuego in the extreme south of Chile, where scrub typhus cases were identified since 2020 [5]. In Cochamó, Los Lagos Region, which has not been sampled before, we collected *H*. *eloisae*, *P*. *neuquenensis*, *Q*. *chiloensis*, and *A*. *expansus*. Of 213 *P*. *neuquenensis* samples, 15 (7.0%) were *Orientia* positive; however, seven of those were collected from rodents co-infested with *H*. *eloisae*, which were *Orientia*-positive. Also two of 43 (4.7%) *A*. *expansus* specimens contained *Orientia* DNA, both originated from rodents co-infested with *Orientia*-positive *H*. *eloisae*. This new finding could indicate a broader spectrum of vector species of *Orientia* spp. in Chile. However, the data have to be interpreted cautiously and require further studies. From Asia, it has been suggested that *Orientia* infection may be transmitted passively between co-feeding chiggers [12,43,49]. This phenomenon, which has also been described for *Rickettsia* spp. in other arthropods [50], might lead to the detection of *Orientia* in mite species that do not serve as natural vector/reservoir. For *O*. *tsutsugamushi*, this has also been proven in animal experiments as well as suggested in field studies from Thailand [51,52]. In Cochamó and Chiloé Island, co-infestation with more than one trombiculid species occurred in >60% of rodents (Table 2). Another possible explanation could be the ingestion of *Orientia* DNA through tissue fluids of infected rodents, since in a previous study tissue samples of 34% of rodents from Chiloé Island tested positive for *Orientia* DNA [53]. *Orientia* infection rates among chigger specimens from Cochamó and Chiloé Island (i.e. 0.3%–7.4%) were similar to those reported in Asia-Pacific (from 0.6%–5%); the rate in Tortel (31.2%), however, was six times higher than the maximum value reported in a recent review [12]. As reported recently, infection rates in samples collected from the environment (6/18, 33.3%) and from humans (10/22, 45.4%) were similarly high [18], indicating that Tortel represents a “hot spot” area for scrub typhus.

The present investigation improves our knowledge on the eco-epidemiology of *Orientia* and its vectors in southern Chile, providing new data on the rodent-associated mite fauna and abundance in different scrub typhus endemic regions. Two species of *Herpetacarus*, *H*. *antarctica* and *H*. *eloisae*, were infected with *Orientia* spp. in all three study areas, which further strengthens their probable role as vectors of scrub typhus. If other mite species, which were found to contain *Orientia* DNA in Cochamó, might also have vector capacity, requires further studies and confirmation. The high density of *Orientia*-infected chiggers in the most southernmost study site highlights that in certain localities there is a high risk to acquire scrub typhus. Outdoor activities such as camping, hiking, fishing, and mountaineering or ecological excursions and wildlife photography are common in southern Chile and associated to scrub typhus cases [19]. The same applies for daily activities or work with contact to vegetation or firewood in rural areas in southern Chile [27]. Therefore, eco-epidemiological studies are not only important to understand the lifecycle of the new Chilean *Orientia* species and its vectors, but also to provide advice for health authorities to prevent and manage cases of scrub typhus in the endemic regions.
